# Rapid Opto-electrochemical Differentiation of Marine Phytoplankton

**DOI:** 10.1021/acsmeasuresciau.2c00017

**Published:** 2022-04-28

**Authors:** Jiahao Yu, Minjun Yang, Christopher Batchelor-McAuley, Samuel Barton, Rosalind E. M. Rickaby, Heather A. Bouman, Richard G. Compton

**Affiliations:** † Physical and Theoretical Chemistry Laboratory, Department of Chemistry University of Oxford, South Parks Road, Oxford OX1 3QZ, Great Britain; ‡ Department of Earth Sciences, University of Oxford, South Parks Road, Oxford OX1 3AN, Great Britain

**Keywords:** marine phytoplankton, electrochemistry, fluoro-electrochemistry, oxidative damage, remote sensing, chlorophyll-a fluorescence

## Abstract

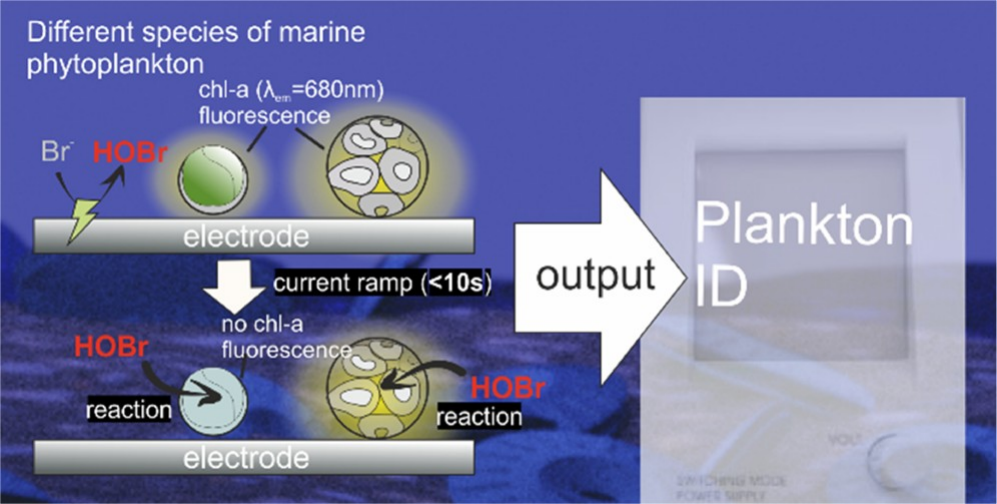

The use of electro-generated oxidants in seawater facilitates the discrimination of different plankton groups via monitoring the decay in real time of their chlorophyll-a (chl-a) fluorescence signals following potentiostatic initiation of electrolysis in their vicinity (YangM.Chem. Sci.2019, 10( (34), ), 7988−79933158833510.1039/c9sc02699aPMC6764473). In this paper, we explore the sensitivity of phytoplankton to different chemical species produced at various potentials in seawater. At low potentials, the oxidation of ca. millimolar bromide naturally present in seawater to hypobromous acid ‘switches-off’ the chl-a signal of individual *Chlamydomonas concordia* cells (green algae) located on the electrode surface within tens of seconds of the potential onset. At higher oxidative potentials, the oxidation of chloride and water produces oxidants (Cl_2_, OH, H_2_O_2_, etc.) that are also lethal to the plankton. To deconvolute the contributions to the response from the chemical identity of the oxidant and the amount of charge delivered to ‘titrate’ the individual living plankton using the loss of fluorescence as the ‘end point’, we introduce a ramped galvanostatic method. This approach enabled the controlled injection of charge applied to a bespoke electrochemical cell in which the plankton are immobilized on an electrode surface for rapid and sensitive measurement. It is shown that the number of moles (charge) of oxidants required to react leading to chl-a switch-off is independent of the chemical identity of the electro-generated oxidant(s) among hypobromous acid, chlorine, or water-derived oxidants. Comparative experiments between *C. concordia* and *Emiliania huxleyi* (where the latter are encapsulated by extracellular plates of calcium carbonate) show that significantly different amounts of absolute charge (moles of electro-generated oxidants) are required in each case to ‘switch-off’ the chl-a signal. The method provides the basis for a tool that could distinguish between different plankton cells within ca. 2 min including the setup time.

## Introduction

Phytoplankton^1^ are aquatic autotrophs that, despite only accounting for less than 1% of photosynthetic biomass, are responsible for half of the world’s primary production and over half of the open-ocean precipitation of CaCO_3_.^2,3^ Fossil records of phytoplankton have suggested their first appearance in the ocean an estimated 10^9^ years ago.^4,5^ In terms of cellular structure, other than polysaccharide-based cell walls, phytoplankton can be found encrusted with elaborate silica shells, plates of calcium carbonate, or hardened organic scales.^6^ It has been suggested that up to approximately 25,000 morphologically defined forms of phytoplankton may exist in the contemporary ocean.^7^ Phytoplankton are often classified into different functional types that are widely used as an organizing principle in biogeochemical models to simulate and project their roles in biogeochemical cycles and climate change.^8^ These functional groups shape the pelagic ecosystem and exert disparate forces on the biological pump. For example, diatoms are the dominant contributor to the export of organic carbon, whereas coccolithophores contribute around half of the open-ocean calcification. These microscopic phytoplankton, ranging from submicron to millimeters in size, are vital for the health of the marine environment owing to their ability to convert light into edible energy via photosynthesis facilitating chlorophyll-a (chl-a) pigments^9^ so forming the basis of the aquatic food web.

The need to identify marine phytoplankton in open-surface water has increased rapidly in the modern era due to an increase in maricultural activities, the desire for an early warning system for frequent recurrence of harmful plankton blooms,^10^ and mapping of marine resources and monitoring effects of climate change on the marine ecosystem. As such, recognizing the importance of species identification because different phytoplankton “functional types” have different impacts on the marine carbon cycle, a massive effort has been put into compiling a comprehensive textbook toward ‘identifying marine phytoplankton.^6^ This extensively documents the characteristic shape, morphology, and structure of the outer cell wall of commonly important species of phytoplankton to aid the identification of the many species via microscopy. Conventional light microscopy relies on structural features that become difficult to routinely detect on smaller cells. Nevertheless, due to the vast diversity and ever-increasing literature reports of new species, the author reflects “the definitive book covering all phytoplankton species in the sea will probably never be realized”.^6^

A systematic and high-throughput alternative approach to distinguish different groups of phytoplankton is to use a flow cytometer.^11,12^ By measuring simultaneously the scattering and fluorescence intensity specific to the type of a phytoplankton cell, the individual populations can be ‘mapped’ and fingerprinted in a two-dimensional plot.^11−14^ In practice, however, a flow cytometer only distinguishes phytoplankton communities with notable size differences because the fluorescence signals of pigments (e.g., chlorophyll and biliproteins) are shared by several taxonomic groups/classes. As the phytoplankton culture ages, the agglomeration of plankton cells and a build-up of detritus and senescent cells result in an ill-defined scattering and fluorescence pattern.^15^ This restricts the laboratory experiments from being conducted almost exclusively in the exponential growth phase of the plankton, limiting its application in the open-ocean environment.^15^ Other laboratory-based molecular techniques such as HPLC^16,17^ and DNA sequencing^18^ can be used to survey ocean basins to obtain a global census of marine plankton diversity.^19,20^

Recently, an electrochemical method provided proof of concept of a new approach to distinguish between the species of marine phytoplankton based on the susceptibility of the plankton cell toward oxidative attack.^21^ By electrochemically generating an oxidative environment at a microsized cylindrical wire electrode,^21,22^ the fluorescence intensities of five different species of phytoplankton in solution, remote from the wire electrode, were seen to ‘switch-off’ as a function of time and location. The time at which the plankton cell ‘switches-off’ is a function of the distance of plankton away from the electrode and its species-specific susceptibility toward oxidative damage generated in situ.^21^ This technique provides a basis for plankton identification as fully developed in the present paper, in particular by using different electrode potentials to generate different oxidant species from seawater and by exploring the amount of charge required to be injected before fluorescence switch-off.

In previous work,^21^ the electrode-generated oxidants from the electrode had to diffuse tens to hundreds of microns to the phytoplankton, requiring a high overpotential (+2.3 V vs Ag wire) to be applied to see measurable oxidation damage of the plankton cell on a realistic experimental timescale of tens of seconds. At this potential, thermodynamically,^23^ a wide range of oxidants can be formed concomitantly in seawater at the carbon wire electrode. This includes, for example, the oxidation of chloride to dichlorine,^24^ bromide to hypobromous acid,^25^ and water to hydrogen peroxide or hydroxide radicals and protons.^26^ The reaction of the oxidants with the phytoplankton results in a sharp drop in the chl-a fluorescence signal after a delay in the onset of the applied potential due to the finite time required for the oxidants to diffuse over tens to hundreds of microns in distance and to penetrate the membranes to reach the chlorophyll in the cell. In the following, attention is focused on the chemical species generated at different potentials and also the amount of charge required for the reaction to be completed. To simplify and speed up the measurement of the latter, a radical experimental redesign was made to minimize the time required for the loss of fluorescence.

The work in this paper shows that a novel simplified approach allows the distinction between plankton via reaction with different electrochemical reagents with the plankton immobilized on an electrode surface of a bespoke novel 3d printed cell. This almost eliminates the variance in the mass-transport time between the electrode and plankton. This novel electrochemical cell geometry has the advantage of giving a near-instant plankton chl-a response due to the very short time required for the electro-generated reactants to diffuse from the electrode to plankton but preserves the group-specific time for quenching.^21^ This thus allows the immediate response of the phytoplankton to oxidants generated in seawater to be investigated as a function of potential with improved resolution. Moreover, due to the optimized bespoke cell geometry, the generator–collector mass transport can be solved mathematically and so allows control over the amount of oxidants, for any plankton species, required to be injected into the phytoplankton cell to switch-off their chl-a fluorescence. The proof-of-concept experiment is then reduced in practice by using galvanostatic-controlled techniques, and we show that, by changing the current applied, the total moles of oxidants required to switch-off the chl-a signal of the same plankton species are independent of the chemical species of oxidants used from within those studied but the amount of charge is species-dependent. Interestingly, it is revealed that although bromide is present in seawater at only millimolar concentration,^27^ much less than chloride (0.56 M) or water (55 M), the oxidation of bromide concentrations at such low levels is still sufficient for the plankton fluorescence to be extinguished.

## Experimental Section

### Chemicals

Bromine water was supplied by Scientific Laboratory Supplies. All other chemicals were supplied by Sigma-Aldrich. All chemicals were of analytical standard and applied without further purification. Ultrapure water (Millipore, resistivity 18.2 MΩ cm at 25 °C) was used to make aqueous solutions, including synthetic ocean water.

### Phytoplankton Cultures

*C. concordia* (marine green alga, RCC 1) and *Emiliania huxleyi* (coccolithophore, RCC 1216) strains were supplied by the Roscoff Culture Collection (RCC), France. Stock cultures of both were maintained by regular subculturing into a fresh growth medium under sterile conditions and during exponential population growth. We used an Aquil synthetic ocean water recipe in place of natural seawater, with F/2 enrichment for RCC 1 and K/2 enrichment for RCC 1216 (see the Supporting Information, Section 1). Both cultures were kept under a 14:10 h light–dark cycle with a PAR intensity of 20–40 μmol m^–2^ s^–1^ at 17 °C, in a PHCbi MLR-352-PE Incubator (PHC Europe B.V.). Centrifugation at a rate of 0.5–1 × 10^3^ RPM and for a duration of ca. 5–8 min was used to pre-concentrate the plankton cell prior to the electrochemical experiments.

### Optics and Image Analysis

The fluorescent light source was supplied by a lighting device HXP 120 V (Carl Zeiss Ltd., Cambridge U.K.). Measurements were made on a Zeiss Axio Examiner, A1 Epifluorescence microscope (Carl Zeiss Ltd., Cambridge U.K.), and an openFrame Inverted microscope (Cairn Research, U.K.), using a 20× air objective (NA = 0.5, EC Plan-Neofluar). The excitation filter was from Thorlabs (FITC 475 ± 35 nm); the dichromic mirror and emission filter were from Zeiss filter set 15 that transmit emission wavelengths above 590 nm. The fluorescence image stacks were collected by a Hamamatsu ORCA-Flash 4.0 digital CMOS camera (Hamamatsu, Japan), providing 16-bit images with a resolution of 4 megapixels, and FLIR BFS-U3-88S6M-C (FLIR Integrated Imaging Solutions, Canada), providing 8-bit images with a resolution of 8.6 megapixels. The temporal evolution of the integrated chl-a intensity for each individual plankton cell was analyzed using ImageJ free-ware (Fiji distribution).

### 3D Printed Cell Setup

**Figure 1:**
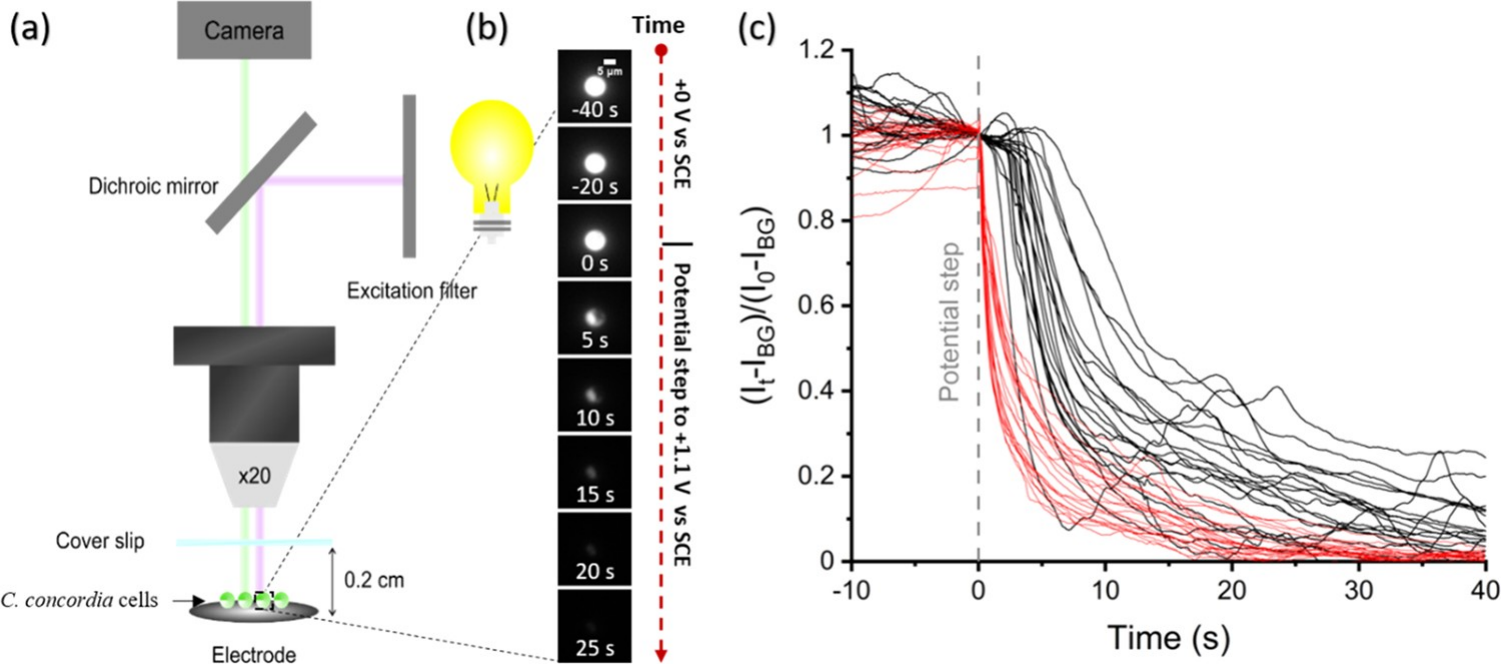
(a) Schematic diagram of the experimental setup. Phytoplankton is stationary on the surface of a glassy-carbon macroelectrode (*r* = 3 mm). Chl-a fluorescence imaging is obtained using λ_ex_ = 475 ± 35 nm and λ_em_ > 590 nm. (b) Real-time images of a living *C. concordia* cell during the opto-electrochemical experiment. The electrolyte is an F/2 medium. The fluorescence intensity of the *C. concordia* cell is displayed in a gray scale. At time zero, the applied potential is stepped from +0 to +1.1 V vs sat. calomel electrode. Scale bar = 5 μm. (c) Plot of the normalized chl-a fluorescence intensity (*I*_t_) integrated over the entire plankton cell against the time. At time zero, the applied potential is stepped from 0 to 1.4 V (red lines) or 1.1 V (black lines). Each line represents an individual *C. concordia* cell. The normalization is background (*I*_BG_)-subtracted integrated intensity (*I*_t_ - *I*_BG_) against that measured at the time of the potential step (*I*_0_ - *I*_BG_).

The bespoke opto-electrochemical cell was designed digitally in Fusion 360 (Autodesk) and was subsequently printed using a Form2 3D-printer equipped with white resin (Formlabs). The base of the opto-electrochemical cell has dimensions (7.5 × 2.5 × 1 cm^3^) similar to that of a glass slide and is suitable to be used under most conventional microscopes. The 3D printed opto-electrochemical cell^28^ hosts a reference electrode (RE-2BP, saturated calomel electrode (SCE), ALS, Japan), graphite counter rod, and a glassy-carbon working electrode (3 mm diameter, MF-2012, BASi). The working electrode is inserted bottom-up into the opto-electrochemical cell with the surface of the electrode facing the objective lens of a conventional up-right microscope, a schematic of which is depicted in Figure 1a. The reference and counter electrodes were inserted into the cell at an acute angle to the objective lens and out of view. A schematic of the opto-electrochemical cell showing the three-electrode setup is reported elsewhere.^28^

Concentrated phytoplankton cells from the culture medium were drop-casted onto the surface of the working electrode. The plankton cells were allowed to sediment onto the electrode under gravity for 2 min before the excess culture solution was removed from the electrode surface by gently soaking it up with a tissue paper. Roughly, 1.5 mL of the F/2 electrolyte was then slowly injected into the reaction chamber to immerse the three-electrode system and fill up the cell. Most of the phytoplankton cells were seen to remain on the surface of the working electrode. A glass slide was then placed at the top to seal the opto-electrochemical cell to leave at least 1 mm of electrolyte solution in between the working electrode and the coverslip.^28^ Potentiostatic control and synchronization with the camera were provided by a previously developed in-house built device and current amplifier (Keithley 427) from Keithley Instruments Inc. In other experiments, a galvanostat as opposed to the potentiostat was used, the principal feature being that a galvanostat controls the electrode current while allowing measurement of the applied potential as a function of time. The galvanostat was home-built and provided a controllable constant current ranging from 1 to 1000 μA and also a ramping current that changed with the time. Both the potentiostat and galvanostat were interfaced with and controlled by a National Instruments USB-6003 device. This digital/analog interface device was controlled by a custom Python script enabling synchronization of the electrochemical and optical measurements.

## Results and Discussion

In the following, we first investigate the response of the marine green algae *C. concordia* to different chemical species formed via electro-oxidation of seawater at different potentials. This is realized by monitoring their cellular chl-a fluorescence signals. Next, the identity of the electro-generated oxidant responsible for the chl-a switch-off in ‘seawater’ is investigated and discussed as a function of potential. We then report proof of concept of a new technique to allow rapid screening of the phytoplankton species in seawater by the use of a galvanostat,^29,30^ which controls the applied current. The resulting generator–collector mass-transport problem is solved analytically to estimate the charge required to be injected and to extinguish the fluorescence of the phytoplankton of interest. Moreover, we further demonstrate that a novel linear current ramping technique in the galvanostatic method is superior to a constant applied current for distinguishing between the phytoplankton species *C. concordia* and *E. huxleyi* with different susceptibilities toward electro-induced oxidative damage. High-resolution microscopy images of *C. concordia*(31) and *E. huxleyi*(28,32) can be found in the Roscoff Culture Collection and elsewhere in the literature.

### Electrochemically Induced Oxidative Stress

Figure 1a is a schematic diagram depicting the opto-electrochemical experiment. Initially, the *C. concordia* cell is stationary on the surface of the electrode and bathed in the artificial seawater culture medium. The electrode surface acts as both a supporting substrate and an electrochemical generator. The average measured cellular radius of *C. concordia* is 3.6 (±0.5) μm. The phytoplankton cell is exposed to continuous fluorescence excitation starting 40 s before a step in the applied potential from a potential of zero Faradaic currents to a selected potential versus SCE was imposed by a potentiostat. Potentials close to 0 V were found to correspond to zero currents. Illustrative results are shown in Figure 1b via a series of images showing transients obtained by optically monitoring the chl-a autofluorescence of a representative *C. concordia* cell experiencing oxidative stress induced by the electrochemical processes from the onset of the step change in applied potential. Within tens of seconds after a step change in the applied potential to 1.1 V, the chl-a intensity of the plankton cell is seen to drop rapidly and eventually becomes indistinguishable from the background. Figure 1c shows the integrated chl-a fluorescence intensity of the plankton cell during the course of the experiment. The integrated intensity presented has been background-corrected and normalized to that measured prior to the application of the potential. Each line shown is the chl-a response from an individual *C. concordia* cell. The black lines are those measured with a step change in the applied potential to +1.1 V vs SCE and the red lines are a further experiment but with a potential of +1.4 V applied. At a lower applied potential of +1.1 V, the chl-a fluorescence intensity of the *C. concordia* is seen to decrease abruptly after 5 s before it tails off to zero. At a higher step potential of +1.4 V, the chl-a intensity is seen to switch-off almost immediately after application of the potential.

It is clear from Figure 1a that the phytoplankton chl-a responds directly to the change in the applied potential, as can be seen for the transient showing steps to +1.4 V vs SCE. This, ultimately, allows investigation of the response of phytoplankton to different oxidants, and the effect of concentrations, available to be electrochemically generated in situ of the artificial seawater culture medium.

**Figure 2:**
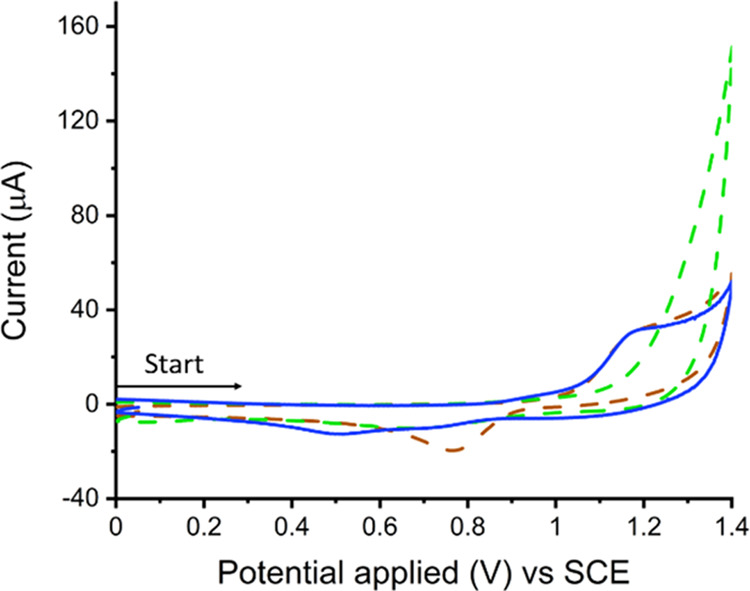
Cyclic voltammograms recorded on a glassy-carbon electrode (radius = 3 mm) in various electrolyte solutions. Solid blue line: seawater-mimicking culture medium F/2. Dotted brown line: 0.42 M NaCl with 0.84 mM bromide ions. Dotted green line: 0.42 M NaCl. Voltage scan rate = 0.1 V s^–1^.

Figure 2 shows a cyclic voltammogram obtained using the above-mentioned glassy-carbon electrode measured in the culture medium, F/2, in the absence of any *C. concordia*. The F/2 culture medium contains chemical constituents (see Table S1) representative of those found naturally in seawater with pH buffered at 8.2. Examples of the ions include metal cations (Na^2+^, Mg^2+^, Ca^2+^, K^+^), halides (Cl^–^, Br^–^, F^–^), bicarbonate (HCO_3_^–^), and various other trace metal ions. In particular, Cl^–^ is present at a concentration of 0.56 M, whereas the concentration of Br^–^ is 0.84 mM. As can be seen in Figure 2, in the F/2 medium (blue line), an irreversible oxidative peak with a peak potential at +1.15 V is evident and a peak current approaching 31.1 μA is seen. Also shown in Figure 2 are voltammograms measured in an electrolyte solution containing only 0.42 M NaCl (green line), and separately, 0.42 M NaCl and 0.84 mM Br^–^ (brown line). It is clear that the cathodic peak seen +1.15 V vs SCE is associated with the oxidation of Br^–^. Figure S1 shows a Pourbaix diagram indicating that the oxidation of bromide to hypobromous acid (HOBr/OBr^–^) is thermodynamically favorable at 1.2 V vs SHE in a pH 8.2 aqueous solution.
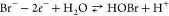
1

Note that in the absence of Br^–^ (solution containing only 0.42 M NaCl), Figure 2 green dotted line, a sharp increase in current is seen near +1.4 V vs SCE. This is due to oxidation of chloride or water near the potential of solvent break down. When bromide is present in the solution, this sharp increase in current associated with oxidation of chloride/water is not seen. This is suggested to be possible because the intermediate Br is adsorbed on the surface of the electrode and ‘inhibits’ the oxidation of Cl^–^ and/or H_2_O, which would otherwise occur at these higher potentials. Having ‘fingerprinted’ the oxidants generated in the F/2 medium at low applied potentials of +1.1 V vs SCE. We next investigate more closely the minimum potentials needed to impact the chl-a response of *C. concordia* in the presence and in the absence of bromide.

**Figure 3:**
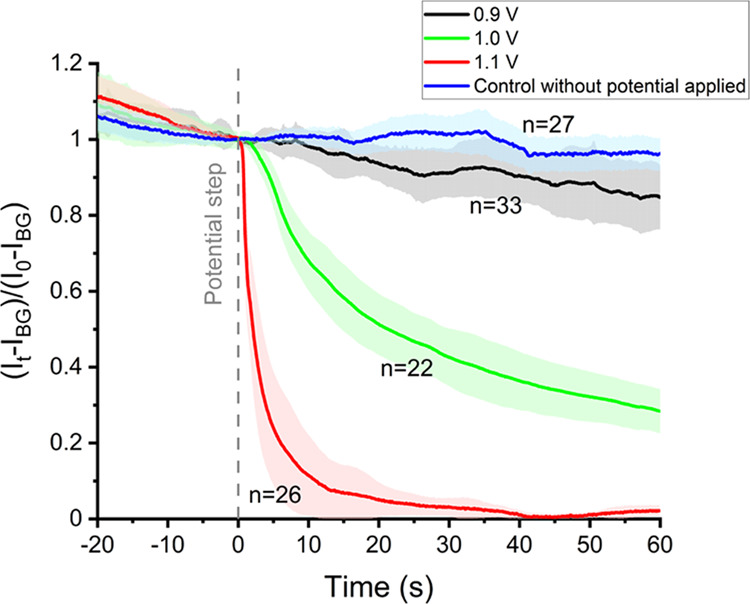
Average chl-a fluorescence of *C. concordia* in response to different steps in the applied potentials at *t* = 0 s. Black line = 0.9 V, green line = 1.0 V, and red line = 1.1 V vs SCE. The blue line is a control experiment in which the opto-electrochemical cell is disconnected from the potentiostat during the course of the experiment. The integral of the chl-a fluorescence intensity (*I*_t_) across the plankton cell over the course of experiments is normalized against that measured at *t* = 0 s after background correction (*I*_BG_). The electrolyte is the F/2 culture medium.

Figure 3 shows the average *C. concordia* chl-a fluorescence intensity transient as a function of time during the same experimental conditions as Figure 1 but for values of the potential applied from *t* = 0 of either +0.9, 1.0, or 1.1 V vs SCE. In the absence of any applied potential, the blue line, the natural decay of the *C. concordia* fluorescence signal remains approximately constant over time. Minor effects were seen when a potential of +0.9 V vs SCE was applied from *t* = 0. At the higher applied potentials, +1.0 or +1.1 V, the chl-a fluorescence of *C. concordia* shows an appreciable deviation from its natural chl-a decay upon exposure to strong ultraviolet (UV) light. It is apparent that the threshold potential required to switch-off the chl-a fluorescence of *C. concordia* is near +1.0 V vs SCE. This is in excellent agreement with the voltammograms shown in Figure 2 where the potential required to drive the oxidation of Br^–^ to form HOBr is near 1.0 V vs SCE. The effect of acid versus oxidants was investigated in our previous work.^21^Figure S2 shows the fluorometer response of the *C. concordia* exposed to submillimolar of bromine water. The pH of the bromine water was pre-adjusted to the pH of seawater, pH 8.2, to form a mixture of HOBr and its conjugate base. Almost immediately after the addition of submillimolar of HOBr, a catastrophic drop in the *C. concordia* chl-a fluorescence intensity was measured, which is in excellent agreement with that seen via in situ electrochemical generations.

Separate opto-electrochemical experiments were carried out in pure water with the addition of 0.48 M KNO_3_ (an inert electrolyte) and in pure water with 0.48 M chloride media, as reported in the Supporting Information. In the absence of bromide, the oxidation of chloride and water at higher cathodic potentials also results in a similar chl-a switch-off behavior. Threshold potentials of 1.2 and 1.4 V were observed for chloride and water, respectively, which are in excellent agreement with the oxidation potentials observed in their respective cyclic voltammograms shown in Figure S3.

It is clear that different oxidants formed at the electrode interface can lead to a switching-off of the plankton chl-a fluorescence and that different oxidants can be formed at varying potentials arising from oxidation of bromide, chloride, or water. At this point, we pose and answer the question, is the switch-off of the plankton fluorescence sensitive to the oxidative species electro-generated in seawater or is the charge required to be ‘injected’ in the plankton cell to observe the chl-a switch-off the same regardless of the species of oxidants? To answer this, we next change the methodology from potentiostat-controlled experiments to galvanostatic control.

### Galvanostatic Experiments: Constant-Current Measurements

A galvanostat alters the potential difference between the working and the counter electrode to control and monitor the current passing through the working electrode.^29,30^ This has two advantages: first, a reference electrode is not essential in a galvanostatic experiment and, in the context of plankton measurements, may increase the longevity of the method in real seawater measurements by mitigating the possibility of biofouling of the reference electrode. Second, the current, and hence the total charge injected from the electrode interface is defined by the user allowing control of the absolute number of moles of oxidative species injected electrochemically to react with the phytoplankton of interest, as discussed below.

**Figure 4:**
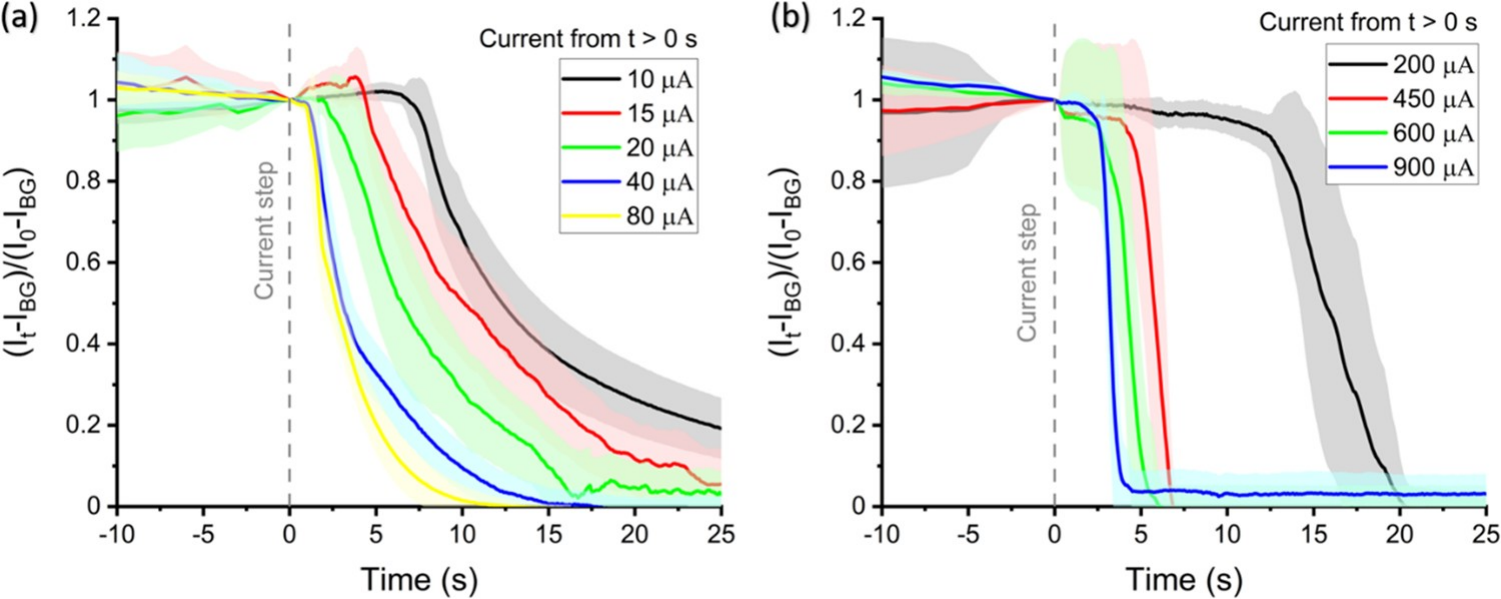
Average fluorescence intensity of two species of phytoplankton at different applied constant currents: (a) *C. concordia* and (b) *E. huxleyi*. For *t* < 0 s, a constant current of 0 A was applied to the working electrode for 40 s. From *t* = 0 s, the applied current is stepped to that shown for the remainder of the experiment. The chl-a fluorescence intensity was monitored throughout the entire experiment. The F/2 culture medium was used as the electrolyte.

Figure 4 shows the average integrated chl-a fluorescence intensity for two species of marine phytoplankton in a constant current galvanostatic experiment. In Figure 4a, at time *t* = 0 s, the current steps from 0 A to values ranging from 10 to 80 μA for *C. concordia*. The potentials the galvanostat required to drive to satisfy these currents values are shown in the Supporting Information, Section 4. Interestingly, under a low constant current of 10 μA, the normalized chl-a transients remain unchanged from unity for ca. 10 s before a large drop is seen. By increasing the applied constant current, this ‘delay’ in the catastrophic drop in chl-a intensity is shortened. Note that this ‘delay’ in the onset of the switch-off is not associated with the time required for the galvanostat to reach the above-mentioned threshold potential to form oxidants, notably oxidation of bromide to hypobromous acid. Figure S5 shows that for the lowest current settings, 10 μA, within ∼2s, the potential applied was ramped up to 1.0 V vs SCE, which is a sufficient potential to form hypobromous acid.

Figure 4b shows the chl-a transients for a different species of phytoplankton, *E. huxleyi*. This species of *E. huxleyi* is encrusted with plates of biogenic CaCO_3_^22,32^ as part of its defense mechanism against grazing among other proposed properties and it also has four membranes surrounding the chloroplast in comparison to the two of *C. concordia*.^33^ As can be seen, it requires a much higher constant current, ranging from 200 to 900 μA, to drive the chl-a switch-off within the same experimental timescale as that seen for *C. concordia*. As the constant current increased from 200 to 900 μA, the onset time of the catastrophic drop in chl-a fluorescence reduces similar to that seen with *C. concordia*.

To investigate the susceptibility of the plankton cell to different species of electro-generated oxidants, we next calculate the total charge that is required to be ‘injected’ into the plankton cell to observe a chl-a switch-off for different applied current densities. Since for high currents, the potential required to be driven by the galvanostat to ‘satisfy’ the current is generally higher, the species of the oxidants formed at the electrode interface as a function of time are necessarily different. Figure S5 shows the potential driven by the galvanostat as a function of time for different applied constant currents. For example, under a constant current of 10 μA, the potential is kept to below 1.2 V, and therefore, the electro-generated oxidant is solely hypobromous acid via oxidation of bromide. For 80 μA, however, the potential is driven to >1.6 V, which is sufficient to drive concomitantly all of the above-discussed oxidation processes (bromide, chloride, water). The galvanostatic methods, in comparison to the potentiostatic method above, have the advantage of mitigating the problem of inhibition of oxidation of Cl^–^ and H_2_O in seawater with the presence of submillimolar of Br^–^ (as discussed above, due to surface adsorption of Br.) because the galvanostat can adjust and drive a higher overpotential to ensure that the flux of oxidants produced satisfies the user-defined current.

For constant-current electrolysis, the concentration of the electro-generated product formed at the electrode interface is given by the Sand equation
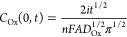
2where *C*_Ox_(0, *t*) is the concentration of the oxidant formed at the electrode interface at time *t* (s), *i* is the user-defined constant current (A), *n* is the number of electrons transferred, *F* is the Faraday constant (C/mol), *A* is the geometric area of the electrode (m^2^), and *D*_Ox_ is the diffusion coefficient of the oxidant (m^2^ s^–1^). Note that eq 2 is derived for a simple one redox-couple system
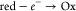
3and eq 2 is valid as long as the flux of species (Red) to the electrode is sufficient to satisfy the applied current. In practice, in the long-time limit as Red is depleted near the facility of the electrode and the current is no longer satisfied via mass transport, the potential shifts cathodically to drive other electrode processes to satisfy the current. In this study, in the seawater-mimicking culture medium, the species Br^–^, Cl^–^, and H_2_O are present at very different concentrations, 0.8 mM, 0.6 M, and 55 M, respectively. In the Supporting Information, Section 5, we show that, due to the relatively low concentration of bromide (0.8 mM) present in seawater, at a high applied constant current of, for example, 80 μA, the submillimolar bromide near the vicinity of the electrode is consumed within seconds of electrolysis and the potential is required to be driven to >1.4 V to drive the oxidation of chloride and water (see the Supporting Information, Section 3 for full discussion). We further show in Figure S6 that the time required to fully deplete 0.6 M Cl^–^ across all constant currents outruns the time required to switch-off the chl-a of *C. concordia* cells. Therefore, we conclude that for higher applied constant currents, although bromide is initially oxidized at the electrode, the system quickly turns to oxidation of chloride, or concomitantly oxidation of both chloride and water, to drive chl-a switch-off in the galvanostatic control experiments. Thus, over the range of the currents used in this study, we make an approximation that Red is present in an excess concentration from *t* = 0 and is not depleted during the timescale of the experiment. The interfacial concentration of the oxidant (Ox) can be therefore described by eq 2.

**Figure 5:**
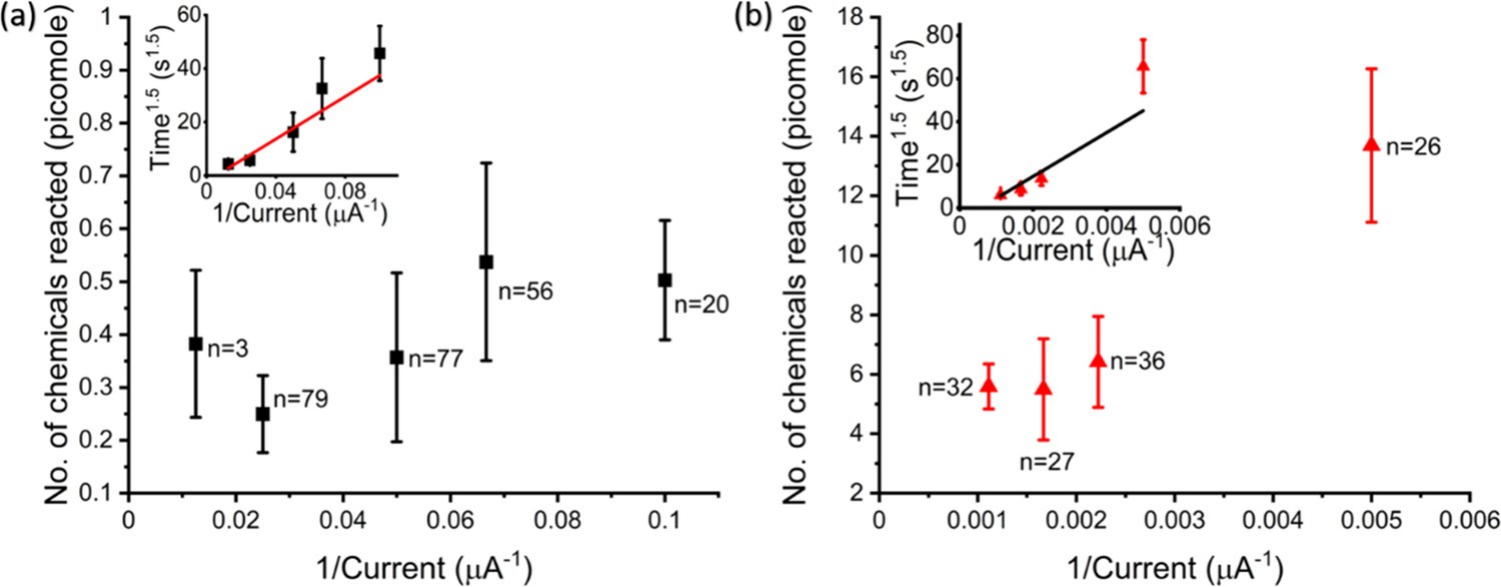
Number of moles of oxidants reacting with different species of phytoplankton during the course of galvanostatic experiments with different user-defined constant currents. (a) *C. concordia* and (b) *E. huxleyi*. The total number of mols is integrated from *t* = 0 s to the time at which the chl-a signal is first dropped to a value below 50% of that measured at *t* = 0.

By substituting the above-discussed Sand equation into the steady-state mass-transport flux to a spherical particle on a plate, the total mol of oxidants that can react with the plankton can be estimated. The resultant expression, shown below as eq 4, shows excellent agreement with that calculated via the fully implicit finite difference method, which is fully discussed in the Supporting Information, Section 5.
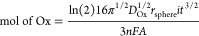
4where *r*_sphere_ is the radius of the plankton cell. Figure 5a shows the total mol of electro-generated oxidants, under mass-transport kinetics, that can react with *C. concordia*. An average *C. concordia* cellular radius of 3.6 (±0.5) μm was used to calculate the moles of reactants. It can be seen that irrespective of the constant current applied, it requires ca. 0.4 picomole of oxidants to react with and extinguish the chl-a fluorescence of the phytoplankton cell. The inlay shows an excellent correlation between *t*^1.5^ and the inverse of the applied current derived in eq 4. The total amount of oxidants required to switch-off the chl-a fluorescence of calcifying *E. huxleyi* (shown in Figure 5b) is approximately 19-fold that calculated for *C. concordia*. An average *E. huxleyi* cellular radius of 3.4 ± (0.2) μm was used in the calculation. It appears that a threshold constant current of at least 450 μA is required for the total number of moles of oxidants required to be injected into the plankton cell to be independent of the constant current. It is likely that for *E. huxleyi*, in contrast to *C. concordia*, the calcified shell has to be dissolved first by a sufficient amount of electro-generated acid, for example, from oxidation of water, before the oxidants can be injected into the cell membrane causing a drop in the chl-a fluorescence signal. Alternatively, it may take a greater amount of oxidants to penetrate the additional membranes around the chloroplast. Therefore, the higher constant current implies that a higher threshold potential is required to, for example, oxidize water to form protons and (short-lived) hydroxyl radicals, where, in this case, protons react to first dissolve the calcium carbonate shell, prior to further reaction.

In natural environment sampling, the total amount of oxidants required to switch-off the most vulnerable and the most resilient species is likely to be more than 19-fold. Therefore, in the case where the plankton sample contains a mixture of unknown species, the constant current approach is a rather inefficient method for species separation. In the next section, we demonstrate that a linear ramping in the applied current is a better technique to rapidly distinguish cells in a sample containing a mixture of *C. concordia* and *E. huxleyi*.

### Galvanostatic Experiments: Ramping Current Measurements

**Figure 6:**
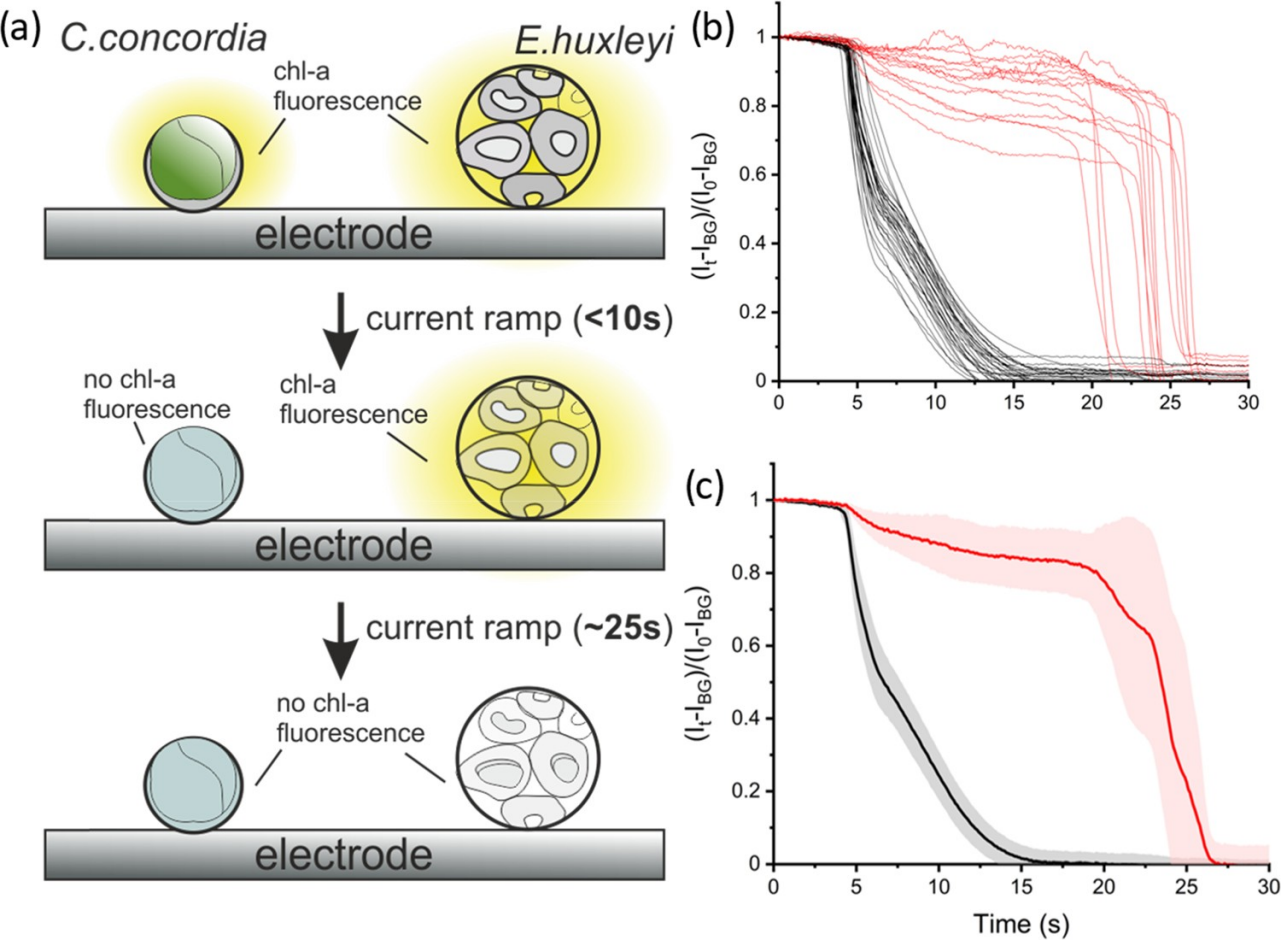
(a) Schematic of the linear current ramp experiment. In this experiment, both *C. concordia* and *E. huxleyi* cells are immobilized onto the electrode surface prior to the experiment. (b) Integrated chl-a fluorescence intensity of individual *C. concordia* (black) and *E. huxleyi* (red) cells during the linear current ramp experiment. The current is ramped from 0 μA at a rate of 10 μA/s from *t* = 0 s. (c) Average integrated chl-a fluorescence intensity of *C. concordia* (black) and *E. huxleyi* (red). The shaded region represents the standard deviation. The F/2 culture medium was used as the electrolyte.

Figure 6a is a schematic diagram showing the concept of a ramping current galvanostat experiment. The two above-studied species of phytoplankton, *C. concordia* and *E. huxleyi*, are present in the same experiment and their chl-a fluorescence intensity is measured as a function of time. Figure 6b shows the cellular chl-a intensity transients for each individual plankton cell with black lines representing *C. concordia* and red lines *E. huxleyi*. The *average* chl-a response of each species is shown in Figure 6c. As can be seen in Figure 6c, the average time for *C. concordia* chl-a intensity to drop to below 50% is 6.6 ± 0.8 s and that for *E. huxleyi* is longer with a time of 23.5 ± 2.1 s. Despite the fact *E. huxleyi* requires *ca* 19 times the total amount of oxidants to switch-off, with the ramping current method, it requires only 4 times longer to differentiate the two species since a higher charge is injected later in the ramp.

For galvanostatic experiments with a linear ramp in current, *i*(*t*) = β*t*, the number of moles of oxidants that can react with a spherical particle on a plate is given by eq 5. For derivation, see the Supporting information, Section 5.
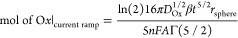
5where β is the rate of current ramp (As^–1^) and Γ(5/2) is the mathematical γ function.

Using eq 5, the maximum total number of moles of reactants that can react with the species to result in a 50% drop in the measured chl-a intensity is 1.0 ± 0.3 and 22.6 ± 4.8 picomoles for *C. concordia* and *E. huxleyi*, respectively. Note that the small discrepancies seen between the number of moles of oxidants calculated in the linear ramping versus that in the constant current technique likely reflect a non-mass-transport controlled rate of reaction between the oxidants and the plankton. This, however, does not impact the merit of this approach as it provides, quantitatively, an upper limit to rank the susceptibility of the different phytoplankton species toward oxidative damage on an absolute scale. The interpretation of the magnitude of the total moles of oxidants, that could have reacted with the plankton under the given galvanostatic conditions, should be taken with caution as it was derived for mass-transport limits.

## Conclusions

This work reports the chl-a response of two marine phytoplankton to oxidative stress generated using potentiostat and galvanostat electrochemical techniques. The phytoplankton cell is immobilized on an electrode thus allowing the near-instantaneous response of the plankton cell to oxidative damage from chemical species generated at different applied potentials to be investigated.

Using chemical compositions that are representative of seawater, we have identified that a threshold potential of +1.0 V vs SCE is required to electrochemically generate a sufficient amount of hypobromous acid, from oxidation of millimolar bromide in seawater to switch-off the chl-a fluorescence of individual *C. concordia* cells during the experimental timescale of 60 s. At higher applied potentials, the oxidation of chloride and water, which are obviously present in seawater at concentrations much higher than that of bromide, also results in a switch-off in chl-a response of *C. concordia*. From the galvanostatic experiments, we conclude that ca. picomoles of oxidants, irrespective of the chemical speciation under the potential investigated, are required to switch-off individual cells of *C. concordia*. In comparison, *E. huxleyi*, a calcifying coccolithophore encrusted with calcium carbonate plates, requires ca. 19 times more moles of oxidants for the fluorescence to be extinguished compared to *C. concordia*. Furthermore, a ramping current, as opposed to a constant applied current galvanostatic method, was found to be more time-efficient in separating multiple unknown species of phytoplankton with different susceptibilities to oxidative damage within one experiment. Current work is investigating a range of phytoplankton species so as to establish a “susceptibility library”, as suggested in ref (21).

## Supporting Information Available

Chemical composition of the culture medium; Pourbaix diagram: bromine; Oxidation of Cl^–^ and H_2_O; Galvanostatic experiments: voltage–time curves; the Sand equation and mass-transport flux to particles on a plate: derivation and validation (PDF)
