# Supplementary material for: Rapid Opto-electrochemical Differentiation of Marine Phytoplankton
Source: ACS Meas Sci Au. 2022 Apr 28;2(4):342–50. doi: 10.1021/acsmeasuresciau.2c00017 (PMC9836062; doi:10.1021/acsmeasuresciau.2c00017)
Supplement: Supplementary file 1 — tg2c00017_si_001.pdf [file tg2c00017_si_001.pdf]

Supporting Information: Rapid optoelectrochemical differentiation of marine phytoplankton

Jiahao Yu,[a] Minjun Yang,[a] Christopher Batchelor-McAuley,[a] Samuel Barton,[b] Rosalind E.M. Rickaby,[b] Heather A. Bouman,[b] Richard G. Compton[a]*

[a] Mr J. Yu, Dr. M. Yang, Dr. C. Batchelor-McAuley and Prof. R. G. Compton
Physical and Theoretical Chemistry Laboratory, Department of Chemistry
University of Oxford,
South Parks Road, Oxford, OX1 3QZ, Great Britain
E-mail: Richard.Compton@chem.ox.ac.uk

[b] Dr. S. Barton, Prof. R. E. M. Rickaby and Prof. H. A. Bouman
Department of Earth Sciences,
University of Oxford,
South Parks Road, Oxford, OX1 3AN, Great Britain

Table of Contents

Section 1: Chemical composition of culture medium 3

Section 2: Pourbaix diagram: bromine 5

Section 3: Oxidation of Cl^- and H_2O 7

Section 4: Galvanostatic experiments: voltage-time curves....... 9

Section 5: The Sand equation and mass-transport flux to particle on a plate: derivation and validation 10

Derivation: constant current 10

Derivation: ramping current 11

Validation: comparison of analytical expression versus numerical simulation 11

References 17

Section 1: Chemical composition of culture medium

The chemical composition of the medium used for cultivating the phytoplankton species is shown in Table S1. Specifically, the oxidizable species on a carbon electrode present at sufficient concentrations in seawater at potentials near or below water breakdown are 0.84mM Br^- (aq), 0.56M Cl^- (aq) and 55M H_2O (l).

Table S1: A summary of the molar concentration of all the components in the synthetic seawater, F/2 growth medium.

		Molar Concentration in final growth medium (mol dm^{-3})
Synthetic Ocean Water (SOW) based on the Aquil medium recipe [1]	NaCl	4.20×10^{-1}
	Na_2SO_4	2.88×10^{-2}
	KCl	9.39×10^{-3}
	$NaHCO_3$	2.38×10^{-3}
	KBr	8.40×10^{-4}
	H_3BO_3	4.85×10^{-5}
	NaF	7.15×10^{-5}
	$MgCl_2 . 6H_2O$	5.46×10^{-2}
	$CaCl_2.2H_2O$	1.05×10^{-2}
	$SrCl_2.6H_2O$	6.38×10^{-5}
F/2 Medium enrichment[2, 3]	$NaNO_3$	8.82×10^{-4}
	$NaH_2PO_4 \cdot H_2O$	3.62×10^{-5}
	$Na_2SiO_3 \cdot 9H_2O$	1.06×10^{-4}
	$FeCl_3 \cdot 6H_2O$	1.17×10^{-5}
	$Na_2EDTA \cdot 2H_2O$	1.17×10^{-5}
	$CuSO_4 \cdot 5H_2O$	3.93×10^{-8}
	$Na_2MoO_4 \cdot 2H_2O$	2.60×10^{-8}
	$ZnSO_4 \cdot 7H_2O$	7.65×10^{-8}
	$CoCl_2 \cdot 6H_2O$	4.20×10^{-8}
	$MnCl_2 \cdot 4H_2O$	9.10×10^{-7}
	Thiamine. HCl (Vitamin B1)	2.96×10^{-7}
	Biotin (Vitamin H)	2.05×10^{-9}
	Cyanocobalamin (Vitamin B12)	3.69×10^{-10}

K/2 medium enrichment based on the K recipe[1], silica included	Na_2SiO_3.9H2O	2.52 x 10^{-4}
	$NaNO_3$	4.41 x 10^{-4}
	NH_4Cl	2.50 x 10^{-5}
	Na_2 b-glycerophosphate	5.00 x 10^{-6}
	H_2SeO_3	5.00 x 10^{-9}
	$Na_2EDTA.2H_2O$	5.55 x 10^{-5}
	$FeCl_3.6H_2O$	5.85 x 10^{-6}
	$MnCl_2.4H_2O$	4.50 x 10^{-7}
	$ZnSO_4.7H_2O$	4.00 x 10^{-8}
	$CoCl_2.6H_20$	2.10 x 10^{-8}
	$Na_2MoO_4.2H_2O$	1.30 x 10^{-8}
	$CuSO_4.5H_2O$	5.00 x 10^{-9}

Section 2: Pourbaix diagram: bromine

Figure S1 shows a Pourbaix diagram of bromine in an aqueous solution at 25°C. As can be seen, near 1.0V vs SCE (+0.244V vs SHE at 25°C), the thermodynamic product as a result of the oxidation of bromide at pH 8.2 is hypobromous acid (HOBr) and its conjugate base (BrO^-).[4] Since the concentration of bromide in seawater is 0.84 mM, the upper limit of HOBr formed in the vicinity of the electrode interface, assuming equal diffusion coefficients, is 0.84mM.

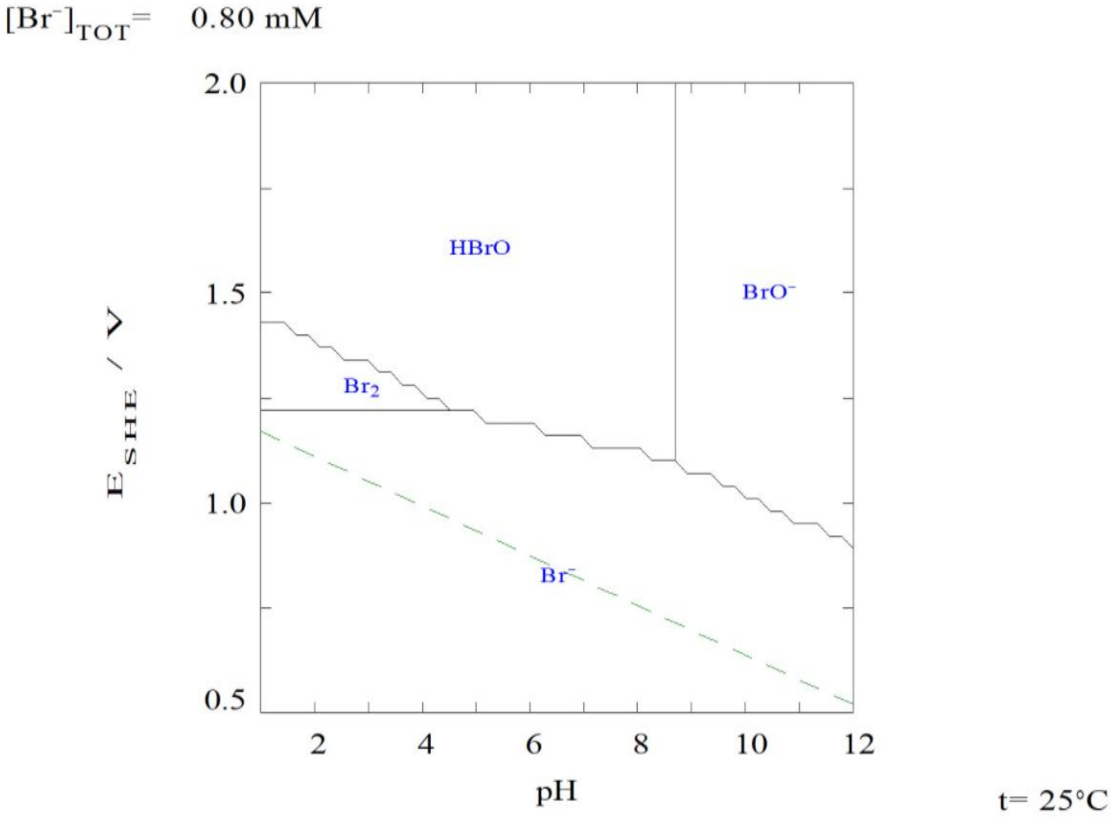

Figure S1. Pourbaix diagram of bromide as a function of pH and potential. Calculated using Hydra/Medusa.[5]

Figure S2 shows the chl-a fluorescence of the *C. concordia* sample measured in a fluorometer after the addition of bromine water. The experimental procedure is reported elsewhere.[6] The bromine water as supplied by the manufacturer is highly acidic with a pH value of 1, to mitigate any pH effects on the chl-a fluorescence of the *C. concordia* sample the bromine water was adjusted to pH 8.2 prior to the experiment. After the

addition of the bromine water, the sample is stirred and the fluorescence signal is measured immediately afterwards with a timescale of tens of seconds of chemical exposure similar to that generated in situ via electrochemistry. As can be seen in Figure S2, addition of pH adjusted bromine water to a final concentration of sub-millimolar (0.42 and 0.84mM) results in a catastrophic drop in the measured chl-a fluorescence of the *C. concordia*. This is in excellent agreement with that observed with in situ electrochemistry.

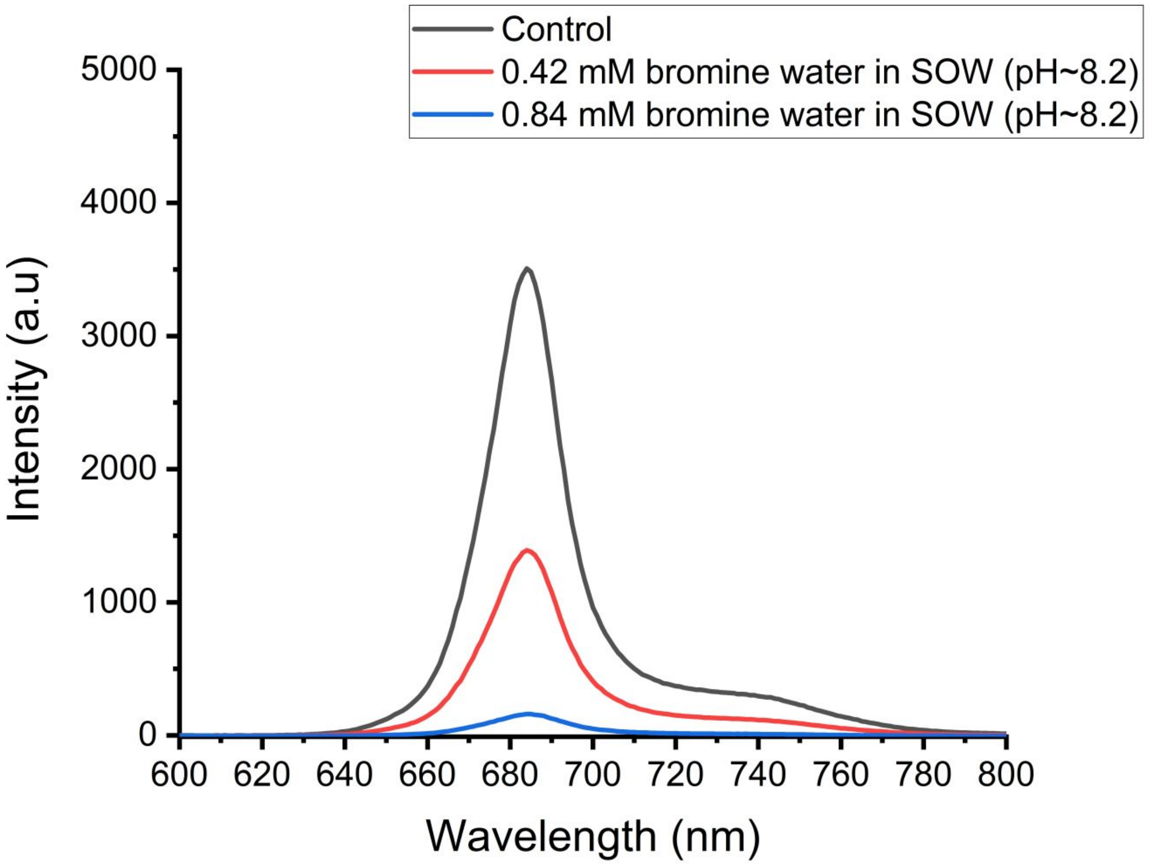

Figure S2. Fluorescence spectrometer emission measurements of C. concordia culture (control in black), and with the addition of 0.42 mM bromine water (in red) and 0.84 mM bromine water (in blue). Excitation wavelength = 430 nm. Scan speed = 2000 nm/min. Excitation and emission bandwidth = 5.0 nm. The pH of the bromine water was adjusted to pH 8.2 prior to the experiment.

Section 3: Oxidation of Cl^- and H_2O

Figure S3 shows the cyclic voltammograms of 0.42M KNO_3(aq) and 0.42M NaCl(aq) recorded on a glassy carbon electrode at a voltage scan rate of 0.1 Vs^{-1}. In the NaCl solution, an exponential rise in current can be seen near +1.2 V. In the KNO_3 solution, however, a higher cathodic potential is required to pass a similar magnitude of current.

In the absence of chloride, in the KNO_3 solution, the only oxidizable species is H_2O resulting in the formation of reactive oxidative species such as $OH^{\cdot}$, H_2O_2 and H^+. In the chloride-containing electrolyte (0.42M KCl(aq)), however, both Cl^- and H_2O can be oxidised.

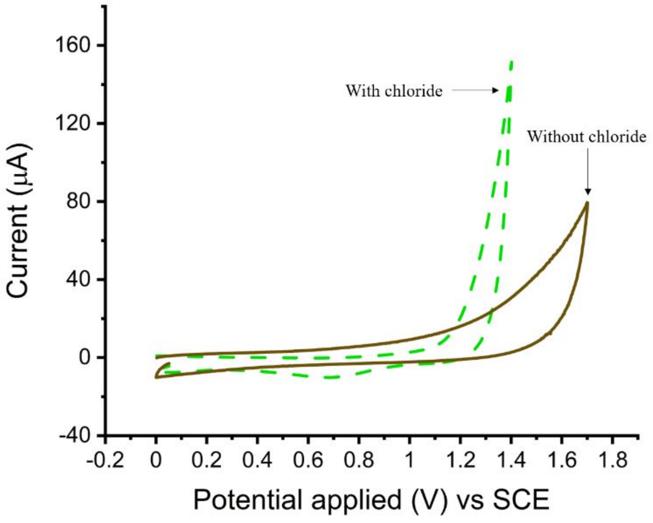

Figure S3. Cyclic voltammograms recorded on a glassy-carbon electrode (radius = 3mm) in various electrolyte solutions. Solid brown line: 0.42M KNO3 in deionised water. Dotted green line: 0.42M NaCl in deionised water. Voltage scan rate = 0.1 V s^{-1}.

Figure S4 shows the average chl-a response of *C. concordia* as a function of potentials in a high-salt electrolyte solution containing a) 0.42M KNO_3 (aq) and b) 0.42M KCl. In the control experiment, the opto-electrochemical cell is disconnected from the potentiostat and the fluorescence signal of the plankton sample is measured as a function of time. As can be seen, in the presence *and* absence of chloride, a different threshold

potential is required to result in a substantial drop in the chl-a intensity over the tens of seconds of the experimental timescale. In both cases, as the potential becomes more cathodic, the time required to switch-off the chl-a fluorescence decreases. In the case of chloride-containing medium (0.42M NaCl (aq)), a threshold potential of +1.2 V vs SCE is required to drive a substantial decrease in the chl-a fluorescence. In the absence of Cl^-, a higher threshold potential of 1.4 V is required. In both cases, the threshold potential is in full consistency with that seen in the respective voltammograms.

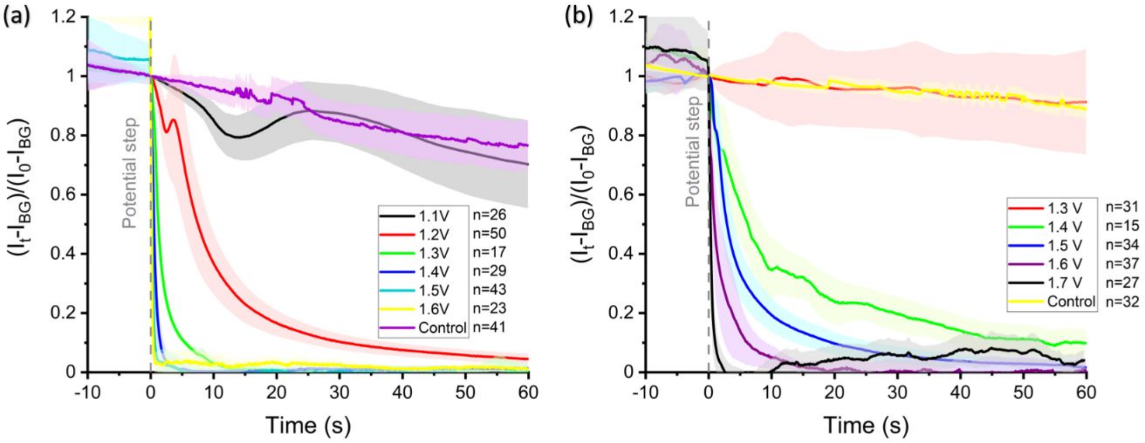

Figure S4. The average chl-a fluorescence of C. concordia in response to different steps in the applied potentials at t = 0 s. (a) Electrolyte: 0.42M NaCl. From time t = 0, the potential was stepped from 0V vs SCE to black line = 1.1 V, red line = 1.2 V, green line = 1.3 V, blue line = 1.4 V, cyan line = 1.5 V and yellow line = 1.6 V vs SCE. Purple line is the control experiment with the opto-electrochemical cell disconnected from the potentiostat. (b) Electrolyte: 0.42M KNO_3. From time t = 0, the potential was stepped from 0V vs SCE to red line = 1.3 V, green line = 1.4 V, blue line = 1.5 V, purple line = 1.6 V and black line = 1.7 V vs SCE. Yellow line is the control experiment with the opto-electrochemical cell disconnected from the potentiostat. The integral of the chl-a fluorescence intensity (I_t) over the plankton cell over the course of experiments is normalised against that measured at t = 0 s after background correction (I_{BG}).

Section 4: Galvanostatic experiments: voltage-time curves

Figure S5 shows the potential the galvanostat applied as a function of time for different constant current settings. The current was stepped from 0 µA from time t = 0 s to values ranging from 10 µA to 900 µA for the remainder of the experiment. The higher the user-defined current, the higher the potential between the working and counter electrode is applied by the galvanostat. For example, it can be seen from Figure S5 b) that approximately +1.1V is driven to obtained 10 µA of current, whereas to reach a higher current of 80 µA a higher potential of ~1.6V was applied by the galvanostat.

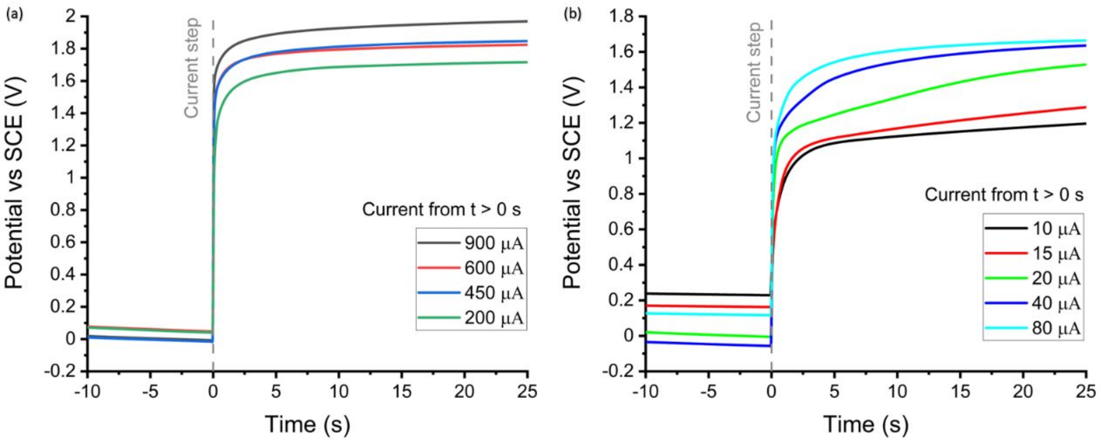

Figure S5. Potential applied vs SCE by the galvanostat as a function of time to maintain the user-defined constant currents: (a) E. huxleyi and (b) C. concordia.

Section 5: The Sand equation and mass-transport flux to particle on a plate: derivation and validation

This section derives the expression used to quantify the absolute mole of charge or oxidants that could have reacted with the plankton under mass-transport flux during the galvanostatic experiments. We first derive an expression for the number of moles of oxidant reacted in the constant current galvanostatic method, followed by ramping current. Next, we validate the analytically derived expressions again a 2D finite difference model. We show that the concentration of chloride is present in excess in the seawater such that it is not depleted during the timescale of the experiment.

Derivation: constant current

The steady-state mass transport flux, J_{MT} (mol s^{-1}), to a spherical particle on a flat surface (aka 'plate') 'bathed' in a homogeneous solution of oxidant (Ox) is[7]

$$J_{MT} = ln\,(2)4\pi D_{Ox}C_{Ox,\,bulk}r_{sphere} \qquad \textit{Equation S1}$$

where r_{sphere} is the radius of the spherical particle (m) on the electrode and $C_{ox,\,bulk}$ is the bulk concentration of the oxidant. Given that the phytoplankton is directly on the electrode and has a radius of 1-10 µm, the bulk concentration that the plankton 'see' can be approximated by the interfacial concentration provided by the Sand equation, shown in Equation 2.[8, 9] Substituting Equation 2 into Equation S1 gives

$$J_{MT} = \frac{d(mol\ of\ ox)}{dt} = \frac{ln\,(2)8\pi D_{Ox}r_{sphere}it^{1/2}}{nFAD_{Ox}^{1/2}\pi^{1/2}} \qquad \textit{Equation S2}$$

Equation S2 shows excellent agreement, where after one second there is no more than 4% deviation to that obtained via finite difference numerical simulation, more of which fully discussed below. Integrating Equation S2 with respect to time gives Equation 4

shown in the main text providing an estimated number of moles of oxidant that reacts with phytoplankton under mass-transport control.

Derivation: ramping current

For galvanostatic experiments with a linear ramp in current, $i(t) = \beta t$, where the β is the rate of current ramp (As^{-1}), the interfacial concentration of oxidants is given by[8, 10]

$$C_{Ox}(0,t) = \frac{2\beta t^{3/2}}{nFAD_{Ox}^{1/2}\Gamma(5/2)} \qquad \textit{Equation S3}$$

where $\Gamma(5/2)$ is the mathematical gamma function. Substituting Equation S3 into Equation S1 gives

$$J_{MT}|_{current\ ramp} = \frac{d(mol\ of\ ox)}{dt} = \frac{ln\,(2)8\pi D_{Ox}^{1/2}\beta t^{3/2} r_{sphere}}{nFA\Gamma(5/2)} \qquad \textit{Equation S4}$$

Integrating Equation S4 with respect to time gives Equation 5 shown in the main text.

Validation: comparison of analytical expression versus numerical simulation

The experiment presented in the main body of the article can be approximately considered as a sphere on a 'plate', where the sphere is the phytoplankton and the supporting plate is the electrode. Here the electrode generates material that is consumed at the particle surface. First, we numerically consider the solution to this mass-transport problem and second, we compare the numerical result to the analytically approximate solution used in the main body of the text.

In this model system species, A is generated at a surface with a fixed current density. Species A can then diffuse away from the surface and is irreversibly consumed at the surface of the particle, where the rate of consumption at the particle surface is simply limited by the rate of mass-transport. Initially there is no species A present in the solution phase. Further, due to the symmetry of the system, this particle on a plate problem only needs to be considered in two dimensions (using cylindrical coordinates).

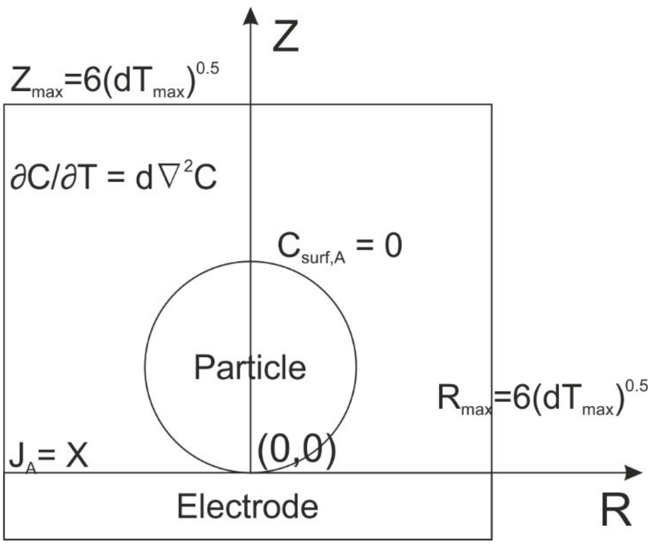

Figure S6 Schematic of the mass-transport problem that needs solving to ascertain the diffusion limited flux to a particle on a generating surface where the flux at the electrode surface is a constant (X). The nomenclate used in this figure follows that used in ref. [11]*Axes are labelled as R and Z and the origin is defined as the point of contact between particle and the electrode surface.*

Figure S4 presents a schematic summary of the differential equation and boundary conditions that needs to be solved for. To numerically solve this diffusion-only problem we use a fully implicit central finite difference scheme.[11] The numerical solution of the resulting system of simultaneous equation was achieved using a GPU optimised iterative solver.[12]

Figure S7 presents a series of example concentration profiles obtained from this simulation, showing how the concentration profile evolves as a function of dimensionless time ($\tau = Dt/r^2$). In this figure, only half of the particle is shown, further, the x and y coordinates are normalised to the size of the particle where the particle has a radius of one. At short times ($\tau = 0.1$) the diffusion layer is small as compared to the size of the particle. As the time increases the diffusion layer increases and at $\tau = 10$ it expands beyond the dimensions of the particle.

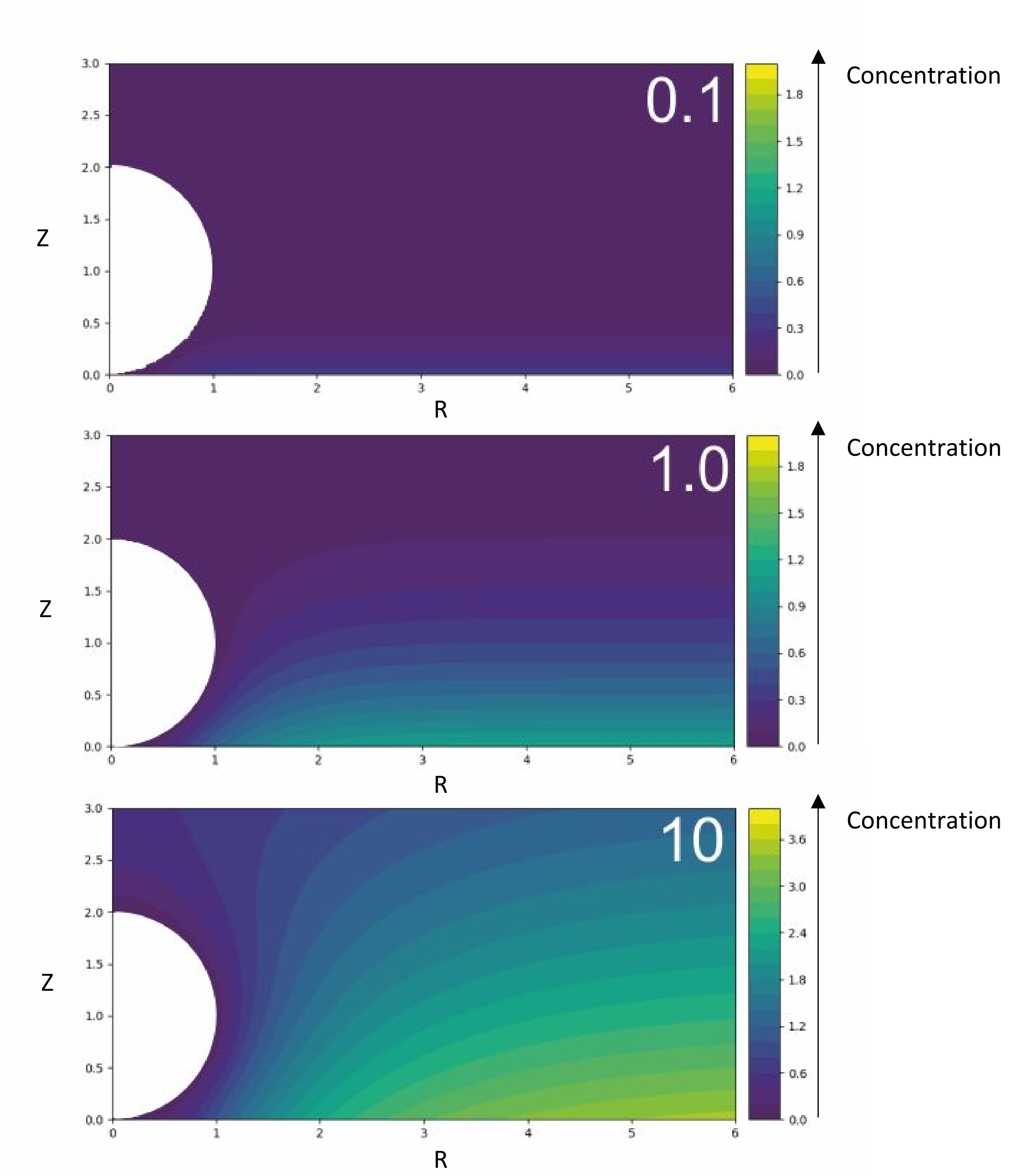

Figure S7. Simulated concentration profiles in the vicinity of a spherical particle at which the species is consumed at a diffusion limited rate. Concentration profiles are presented at three different times (τ = 0.1, 1.0 and 10) after the onset of the applied constant flux at the electrode surface. Note that due to the use of a fixed flux at the electrode surface the concentration of the reagent increases with approximately the square root of time. Axes are labelled as R and Z. The colour scheme corresponds to concentration.

From these concentration profiles it is possible to calculate the expected flux to the particle. Figure S8 presents the dimensionless flux (=j/DCr) of species A to the particle surface as a function of the dimensionless time. Also plotted on the Figure is the flux as predicted from the analytical approximate solution given earlier. Given the simplicity of the approximate model the agreement with the numerically simulated flux is

remarkable. At $\tau = 10$ the simulated flux differs by less than 4% from that predicted analytically. Having shown that the Sand equation matches with the 2D finite difference simulation, we next further demonstrate that the Sand equation is valid throughout the experiment timescale.

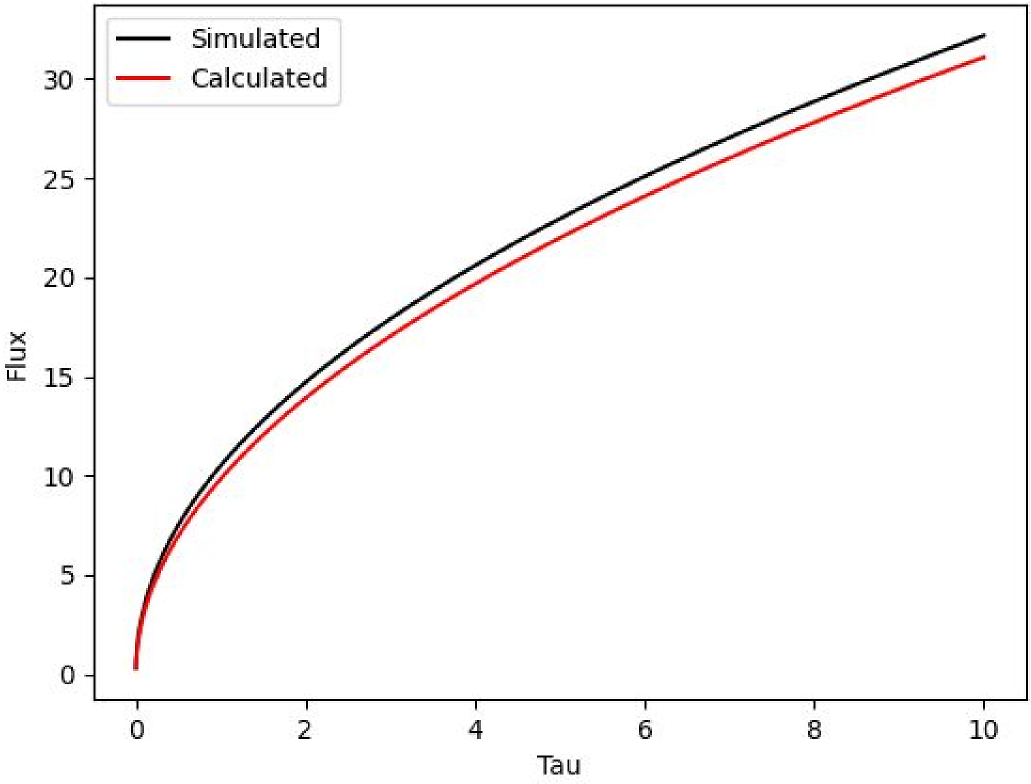

Figure S8. Comparison of the numerically simulated flux (black) with the analytical approximate solution obtained from the modified Sand equation.

The Sand equation[8, 9] is an analytical expression reporting the interfacial concentration of the electrochemically active reactant (red) and product (ox) in a galvanostatic experiment. In practice, however, because bromide exists in seawater at a much lower concentration (0.84mM) than that of chloride (0.56M) and water (55M) the bromide may be depleted at the interface during the galvanostatic experiment. Using the Sand equation for constant current, Equation 2 in the main text, we can estimate the time at which a finite concentration of reactants becomes depleted at the electrode interface.

Figure S9 shows the time required to deplete the seawater concentration of 0.84mM of bromide (brown line) and, shown in the inlay, 0.56M of chloride (green line). Notice the scale of the applied current required to deplete chloride at the electrode interface is much higher than that calculated for bromide. Overlaid as black squares are the average time taken to switch-off the chl-a fluorescence of the *C. concordia* cells during constant current galvanostatic experiments. Red triangles in the inlay are those observed for *E. huxleyi* cells. As can be seen, due to the relatively low concentration of bromide, the depletion of bromide at the electrode interface occurs *before* the switch-off of either of the two phytoplankton species studied. The time required to deplete the interfacial concentration of chloride, however, is longer than that required to switch-off off the chl-a fluorescence of the two phytoplankton species. Therefore, we conclude that in the galvanostatic experiments, in seawater and at low applied currents, initially bromide is oxidised to form oxidants at the electrode interface, it depletes quickly due to the sub-millimolar concentration and it is the oxidants produced by oxidation of chloride (and water at higher cathodic potentials) that is responsible for the chl-a switch off.

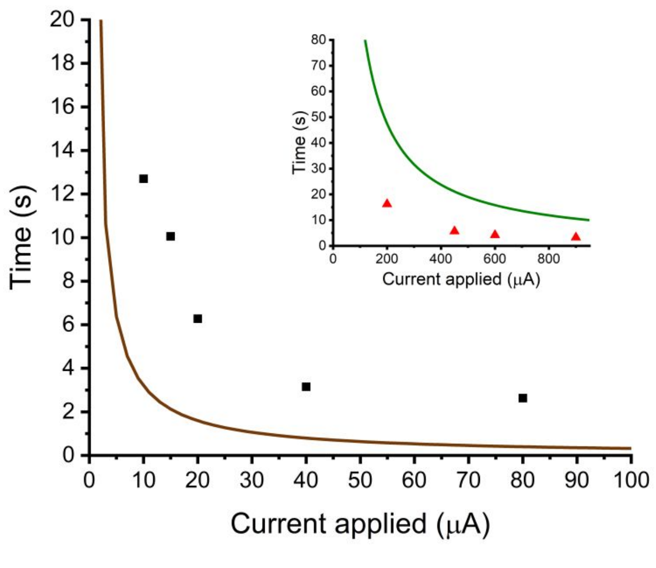

Figure S9. The time needed to deplete interfacial bromide ions (brown line) and chloride ions (green line) as a function of the current estimated using the Sand equation. A diffusion coefficient of 1 x 10^{-9}

m^2s^{-1} was used for the calculation. Time taken to inhibit 50% fluorescence intensity of C. concordia (black square dots) and Emiliania huxleyi (red triangular shapes) is displayed for comparison.

References

1. Morel, F. M.; Rueter, J.; Anderson, D. M.; Guillard, R., AQUIL: A CHEMICALLY DEFINED PHYTOPLANKTON CULTURE MEDIUM FOR TRACE METAL STUDIES 1 2. *Journal of Phycology* **1979,** *15* (2), 135-141.
2. Guillard, R. R., Culture of phytoplankton for feeding marine invertebrates. In *Culture of marine invertebrate animals*, Springer: 1975; pp 29-60.
3. Guillard, R. R.; Ryther, J. H., Studies of marine planktonic diatoms: I. Cyclotella nana Hustedt, and Detonula confervacea (Cleve) Gran. *Canadian journal of microbiology* **1962,** *8* (2), 229-239.
4. Ziderman, I., Variations in the Composition of Bromine Water During Oxidation Reactions. *Israel Journal of Chemistry* **1973,** *11* (1), 7-20.
5. Puigdomenech, I., Hydra/Medusa chemical equilibrium database and plotting software. *KTH Royal Institute of Technology* **2004**.
6. Yang, M.; Batchelor-McAuley, C.; Chen, L.; Guo, Y.; Zhang, Q.; Rickaby, R. E.; Bouman, H. A.; Compton, R. G., Fluoro-electrochemical microscopy reveals group specific differential susceptibility of phytoplankton towards oxidative damage. *Chemical Science* **2019,** *10* (34), 7988-7993.
7. Bobbert, P.; Wind, M.; Vlieger, J., Diffusion to a slowly growing truncated sphere on a substrate. *Physica A: Statistical Mechanics and its Applications* **1987,** *141* (1), 58-72.
8. Bard, A. J.; Faulkner, L. R., *Electrochemical Methods: Fundamentals and Applications, 2nd Edition*. John Wiley & Sons, Incorporated: 2000.
9. Sand, H. J., III. On the concentration at the electrodes in a solution, with special reference to the liberation of hydrogen by electrolysis of a mixture of copper sulphate and sulphuric acid. *The London, Edinburgh, and Dublin Philosophical Magazine and Journal of Science* **1901,** *1* (1), 45-79.
10. Murray, R. W.; Reilley, C. N., Chronopotentiometry with programmed current: II. Response function additivity principles applied to current programming and multicomponent systems. *Journal of Electroanalytical Chemistry (1959)* **1962,** *3* (3), 182-202.
11. Compton, R. G.; Banks, C. E., *Understanding Voltammetry*. World Scientific: Singapore, 2018.
12. Wong, R.; Batchelor-McAuley, C.; Yang, M.; Compton, R. G., The steady-state diffusional flux to isolated square cuboids in solution and supported on an inert substrate. *Journal of Electroanalytical Chemistry* **2021,** *903*, 115818.
